# Identification of hub genes in colorectal cancer based on weighted gene co-expression network analysis and clinical data from The Cancer Genome Atlas

**DOI:** 10.1042/BSR20211280

**Published:** 2021-07-26

**Authors:** Yu Zhang, Jia Luo, Zhe Liu, Xudong Liu, Ying Ma, Bohang Zhang, Yuxuan Chen, Xiaofeng Li, Zhiguo Feng, Ningning Yang, Dayun Feng, Lei Wang, Xinqiang Song

**Affiliations:** 1 College of Life Sciences, Xinyang Normal University, Xinyang 464000, China; 2 Department of Computer Science, City University of Hong Kong, Hong Kong 999077, China; 3 Department of Recovery Medicine, People’s Liberation Army 990 Hospital, Xinyang 464000, Henan, China; 4 Department of Pathology, First Affiliated Hospital of Xi’an Jiaotong University, Xi’an 710061, China; 5 College of Science, Qiongtai Normal University, Haikou 571127, China; 6 Tropical Biodiversity and Bioresource Utilization Laboratory, Qiongtai Normal University, Haikou 571127, China; 7 Department of Neurosurgery, Tangdu Hospital, Air Force Medical University, Xi’an 710038, China; 8 College of Medicine, Xinyang Normal University, Xinyang 464000, China

**Keywords:** Colorectal cancer, Hub gene, Immunohistochemistry, Weighted gene co-expression network analysis

## Abstract

Colorectal cancer (CRC) is one of the most common tumors worldwide and is associated with high mortality. Here we performed bioinformatics analysis, which we validated using immunohistochemistry in order to search for hub genes that might serve as biomarkers or therapeutic targets in CRC. Based on data from The Cancer Genome Atlas (TCGA), we identified 4832 genes differentially expressed between CRC and normal samples (1562 up-regulated and 3270 down-regulated in CRC). Gene ontology (GO) analysis showed that up-regulated genes were enriched mainly in organelle fission, cell cycle regulation, and DNA replication; down-regulated genes were enriched primarily in the regulation of ion transmembrane transport and ion homeostasis. Weighted gene co-expression network analysis (WGCNA) identified eight gene modules that were associated with clinical characteristics of CRC patients, including brown and blue modules that were associated with cancer onset. Analysis of the latter two hub modules revealed the following six hub genes: adhesion G protein-coupled receptor B3 (*BAI3*, also known as *ADGRB3*), cyclin F (*CCNF*), cytoskeleton-associated protein 2 like (*CKAP2L*), diaphanous-related formin 3 (*DIAPH3*), oxysterol binding protein-like 3 (*OSBPL3*), and RERG-like protein (*RERGL*). Expression levels of these hub genes were associated with prognosis, based on Kaplan–Meier survival analysis of data from the Gene Expression Profiling Interactive Analysis database. Immunohistochemistry of CRC tumor tissues confirmed that *OSBPL3* is up-regulated in CRC. Our findings suggest that *CCNF, DIAPH3, OSBPL3*, and *RERGL* may be useful as therapeutic targets against CRC. *BAI3* and *CKAP2L* may be novel biomarkers of the disease.

## Introduction

Colorectal cancer (CRC), which includes colon and rectal cancers, is one of the most common cancers of the digestive system [1]. It is the second leading cause of cancer-related mortality and the third leading cause of cancer-related incidence worldwide [2]. It occurs in three histopathological types, including adenocarcinoma, squamous cell carcinoma, and mucinous carcinoma; adenocarcinoma is the most common type, accounting for ∼95% of all CRC cases [3].

Risk for CRC has been linked to defects in DNA replication and DNA methylation, as well as instability of chromosomes and microsatellites [4–7]. As in many cancers, early stages of CRC appear to involve up-regulation of DNA replication licensing proteins [5]. Up to 15% of CRC cases involve DNA microsatellite instability, which leads to DNA replication errors [8].

Surgery remains the primary method to treat CRC, but the post-surgery recurrence rate is high, the post-surgery 5-year mortality rate is high [9]. In part, this is because most patients with CRC are diagnosed relatively late in the disease [10]. Therefore, it is imperative to understand the molecular mechanism involved in carcinogenesis in order to identify prognostic biomarkers and potential therapeutic targets for CRC.

High-throughput sequencing technologies provide new views into the genomic, transcriptomic, and epigenomic signatures of cancers. Systems biology, especially network methods, can effectively integrate multiple, large-scale datasets of complex human diseases, especially cancer [11–13]. Weighted gene co-expression network analysis (WGCNA), for example, is an efficient, accurate method for extensive multigene analysis [14,15]. The WGCNA package in the R suite is a comprehensive collection of R functions for performing all aspects of weighted correlation network analysis [16]. It can construct a scale-free network to explore the correlation between different genomes or between samples and clinical features [17]. WGCNA has been widely used to identify related clinical modules and hub genes in different types of cancer. For example, one WGCNA study was able to associate the expression of six hub genes with progression of clear human cell renal cell carcinoma and with prognosis of patients [18]. Another WGCNA study drew on data from the Gene Expression Omnibus (GEO) and The Cancer Genome Atlas (TCGA) to identify 15 hub genes as candidate breast cancer biomarkers [19]. A third study used WGCNA to identify four hub genes that may be candidate biomarkers of adrenocortical carcinoma [20].

The present study exploited the power of WGCNA to analyze the pathogenesis of CRC. RNA sequencing data from CRC samples were downloaded from the TCGA, and genes differentially expressed between CRC and normal tissues were analyzed at the expression and functional levels. Functional enrichment of differentially expressed genes (DEGs) was analyzed using Gene Ontology (GO) in the clusterprofiler R package. WGCNA of the DEG matrix identified modules related to clinical characteristics of CRC patients. Hub genes identified through these bioinformatics analyses were verified using survival analysis, immunohistochemistry of CRC tissues, and analysis of the literature. Our findings provide testable hypotheses about genes involved in CRC and, by extension, potential biomarkers and therapeutic targets.

## Materials and methods

### Data sources and pre-processing

**Figure 1:**
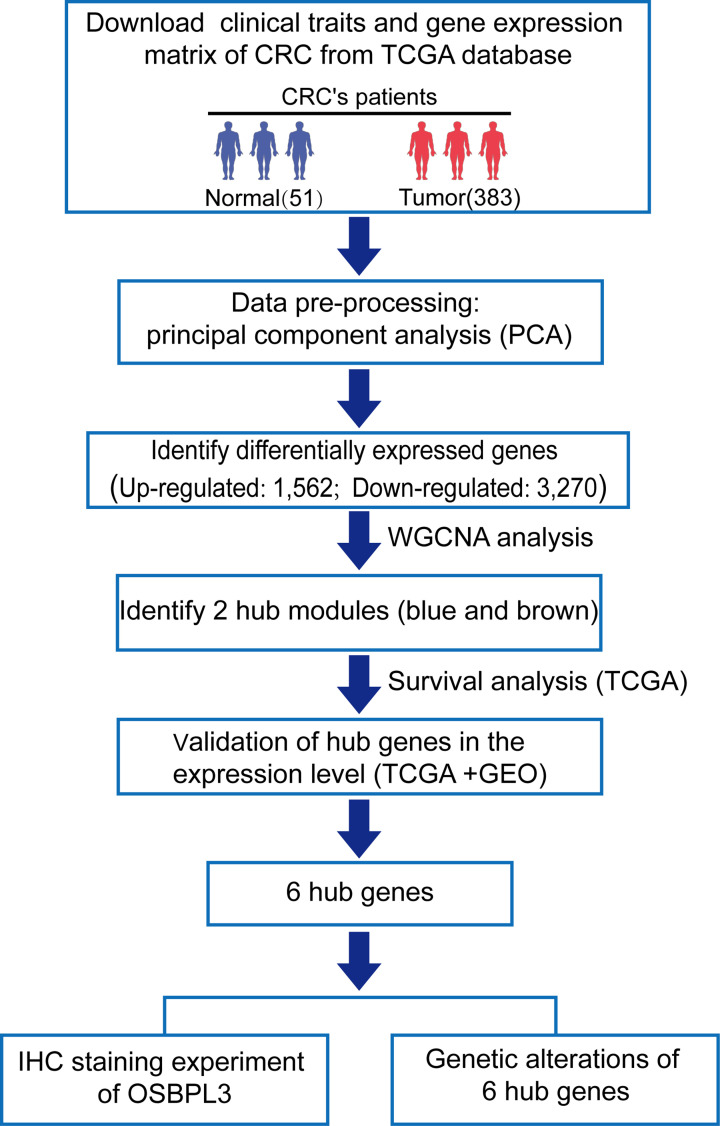
Workflow of searching hub genes in CRC Abbreviation: IHC, immunohistochemistry.

RNA sequencing data and clinical information on patients were downloaded on 22 July 2018 from the ‘Colon and Rectal Cancer’ cohort of TCGA (https://www.cancer.gov/about-nci/organization/ccg/research/structural-genomics/tcga), hosted at the Xena website of the University of California at Santa Cruz [21](http://xena.ucsc.edu/; Table 1 and Supplementary Tables S1 and S2). The RNA sequencing data corresponded to 383 tumor samples and 51 normal tissue samples from 434 CRC patients. We excluded samples if the first two principal components identified through principal component analysis were unable to distinguish tumor tissue from normal tissue. The workflow of the study is shown in Figure 1.

**Table 1:** The clinical information and sample size for TCGA CRC dataset

Characteristics	Alive (*n*=574)	Dead (*n*=156)	Total (*n*=730)	*P*
**Sex**				
Female	274 (47.7)	74 (47.4)	348 (47.7)	
Male	300 (52.3)	82 (52.6)	382 (52.3)	1
**Age, years**				
Mean (SD)	65.6 (12.7)	70.4 (12.7)	66.6 (12.9)	
Median [min, max]	66.5 [31, 90]	73 [34, 90]	68 [31, 90]	
**Body weight**				
Mean (SD)	83.1 (22.8)	74.4 (18.6)	81.1 (22.2)	
Median [min, max]	82.3 [34, 175.3]	71.9 [40, 140]	79.3 [34, 175.3]	
**Cancer type**				
Colon	422 (73.5)	123 (78.8)	545 (74.7)	
Rectum	152 (26.5)	33 (21.2)	185 (25.3)	0.21
**Histological type**				
Colon adenocarcinoma	378 (66.0)	101 (65.2)	479 (65.8)	
Colon mucinous adenocarcinoma	50 (8.7)	20 (12.9)	70 (9.6)	
Rectal adenocarcinoma	132 (23.0)	32 (20.6)	164 (22.5)	
Rectal mucinous adenocarcinoma	13 (2.3)	2 (1.3)	15 (2.1)	0.389
**Stage**				
I	111 (19.8%)	9 (6.2%)	120 (17.0%)	
II	48 (8.6%)	13 (8.9%)	61 (8.6%)	
IIA	172 (30.7%)	25 (17.1%)	197 (27.9%)	
IIB	12 (21.4%)	3 (2.1%)	15 (2.1%)	
III	31 (55.3%)	8 (5.5%)	39 (5.5%)	
IIIA	13 (2.3%)	2 (1.4%)	15 (2.2%)	
IIIB	78 (13.9%)	15 (10.3%)	93 (13.2%)	
IIIC	38 (6.8%)	23 (15.8%)	61 (8.6%)	
IV	37 (6.6%)	41 (28.1%)	78 (11.0%)	
IVA	18 (3.2%)	7 (4.8%)	25 (3.5%)	
IA	1 (0.2%)		1 (0.1%)	
IIC	2 (0.4%)		2 (0.3%)	

Values are *n* (%), unless otherwise noted.

### Identification of CRC DEGs

The ‘limma’ function in the R suite (version: 3.3.3) [22,23] was used to identify DEGs between CRC and normal colorectal tissues. DEGs were defined as those showing |log_2_(fold change) | > 1 and *P*<0.01. Volcano plots of DEGs were plotted using ‘ggplot2’ in R.

### Functional enrichment of DEGs

After converting DEG identifiers using the ‘org.Hs.eg.db’ program (version: 3.10) within R, DEGs were analyzed for functional enrichment based on GO [22] using the ‘clusterProfiler’ program (version: 3.14.3) in R. GO terms with *P*<0.05 were considered statistically significant.

### WGCNA

We used BiocManger (version: 1.30.10) in the R suite to download the WGCNA package (version: 1.70-3) to construct the DEG co-expression network [24,25]. First, the DEG expression matrix was filtered through the goodSamplesGenes function in WGCNA to remove unqualified genes and samples. Second, the flashClust tool in R was used to perform cluster analysis of samples in order to detect outliers. Third, matrices of Pearson correlation coefficients (PCCs) were calculated for pair-wise gene comparisons. Fourth, an appropriate soft threshold power (β) was selected to ensure a scale-free network using the pickSoftThreshold function. Fifth, the adjacency matrix was constructed using the power function

$$a_{\mathrm{ij}}\;=\;|c_{\mathrm{ij}}{|}^{\beta },$$

where c_ij_ refers to the PCC between genes i and j, and a_ij_ refers to adjacency between those two genes. Then, the topological overlap matrix (TOM) was constructed using the adjacency function

$${\mathrm{TOM}}_{i,j}\;=\;\frac{l_{\mathrm{ij}}+\;a_{\mathrm{ij}}}{\min(k_{i}+k_{j})+1-a_{\mathrm{ij}}}$$

where l_ij_ refers to the product’s sum of the adjacency coefficients of the nodes connected by genes i and j, and k refers to the sum of the adjacency coefficients of the given gene with all other genes in the weighted network. The TOM was used to calculate a dissimilarity measure (1-TOM) to allocate genes into modules based on their similar expression [26], using the dynamic tree cutting method [27]. The minimum number of genes in each module was set to 30.

### Selection of clinically significant modules and identification of CRC hub genes

First, principal component analysis was used to describe module eigengenes, corresponding to a single characteristic expression profile across all genes within each module. Correlations between these eigengenes and clinical characteristics were calculated in order to identify which modules were clinically significant. The linear relationship between gene expression and clinical characteristics were assigned a gene significance (GS) equal to the logarithm of the *P*-value for the individual gene. If GS strongly correlated with module membership (MM), defined as the correlation between the module’s eigengenes and individual gene expression profiles, we concluded that the module’s central genes correlated with CRC [28,29]. We considered these central genes as candidate hub genes.

### Bioinformatics validation of hub genes

The expression levels of hub genes in CRC samples were explored using the GEPIA website (http://gepia.cancer-pku.cn/), and the ability of hub genes to predict survival was assessed based on Kaplan–Meier analysis using the ‘survival’ package (version: 3.2-7) in the R suite. First, we obtained DEG expression profiles and prognostic data for 360 CRC tumor samples from the TCGA, then we determined each gene’s median expression value. Samples were assigned to ‘high expression’ or ‘low expression’ groups for a given gene based on whether that gene was expressed at a level higher or lower than the median. Differences in survival between high or low expression groups were assessed for significance using the log-rank test. If this test was associated with *P*<0.05, we considered the gene to be a validated hub gene.

We then screened for differences in hub gene expression between normal and CRC tissues based on colon adenocarcinoma (COAD) and rectal adenocarcinoma (READ) data from the TCGA and the Genotype-Tissue Expression Project (GTEX) on the GEPIA website. Expression levels were normalized by their mean value, and differences associated with *P*<0.01 were considered statistically significant. Hub genes were further validated by analyzing their expression differences between CRC and normal tissues using the ‘ggpubr’ package (version: 0.4.0) in the R suite and the GSE33113 dataset [30,31] in the GEO database (https://www.ncbi.nlm.nih.gov/geo/). Independent-samples *t* test was applied as standard.

We mapped the hub genes’ genome, including mutations, copy number variants (CNVs), and mRNA expression z-scores (RNASeqV2 RSEM) using data from 594 CRC samples from the colorectal adenocarcinoma dataset in the Pan-Cancer Atlas of TCGA. We also used MutationMapper tools to depict the mutation landscape of each hub gene. We accessed and analyzed the data using CBioPortal (http://www.cbioportal.org/).

### Immunohistochemical validation of *OSBPL3* as a hub gene

Immunohistochemistry of tumor and paired normal tissues from three CRC patients from Tangdu Hospital of the Fourth Military Medical University was performed as described [32,33]. Written informed consent for tissue donation, which clearly stated the purpose of our study, was obtained from all of the patients. Tissues were fixed with formalin, embedded in paraffin and sliced into 3-μm-thick sections. After deparaffinization and inactivation of endogenous catalase, the sections were boiled in sodium citrate buffer to expose antigenic sites, then blocked in 5% normal goat serum for 1 h to prevent non-specific binding. Next, the sections were incubated with anti-OSBPL3 antibody (1:50 dilution; Proteintech, 12417-1-AP) at 4°C overnight, and binding was detected using the avidin–biotin–peroxidase method. Sections were counterstained with Hematoxylin. Two experienced researchers independently evaluated the results.

## Results

### Data pre-processing

**Figure 2:**
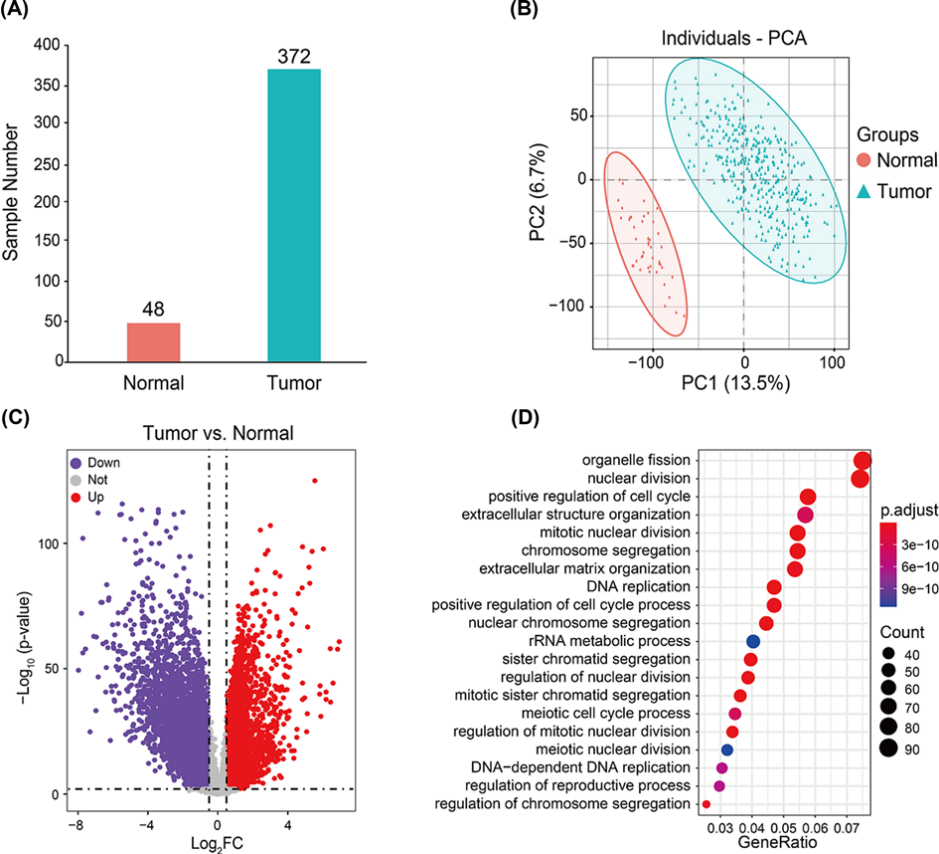
Identification of DEGs between 48 normal and 372 CRC samples (**A**) Principal component analysis. (**B**) Volcano plot. Purple dots represent genes down-regulated in CRC; gray dots, genes not differing significantly between CRC and normal tissues; and red dots, genes up-regulated in CRC. (**C**) **The volcano plot. Purple dots represent down-regulated genes, gray dots represent not significant genes, and red dots represent up-regulated genes.** (**D**) GO analysis of functional enrichment of up-regulated genes. Dot size reflects the number of genes enriched under the given ontology term, and the color indicates the significance of enrichment.

Our expression data came from 51 normal samples and 383 tumor samples (Supplementary Table S1). Filtering based on principal component analysis led to the exclusion of 11 tumors and 3 normal samples from the final dataset (Figure 2A,B). The first two principal components distinguished tumor from normal samples well, accounting for 13.5% (first component) and 6.7% (second component) of the observed differences. The gene expression profiles from these 420 samples were used in subsequent analyses.

### Identification of DEGs in CRC samples and GO enrichment analysis

A total of 4832 DEGs were identified between 48 normal and 372 CRC samples, including 1562 up-regulated and 3270 down-regulated genes (Figure 2C, Supplementary Table S3). To explore the potential biological function of DEGs in CRC, we performed GO enrichment analysis (Supplementary Tables S4 and S5). The up-regulated DEGs were involved mainly in nuclear division, cell cycle regulation, chromosome segregation, and DNA replication (Figure 2D). In contrast, the down-regulated DEGs were involved mainly in the regulation of ion transmembrane transport, muscle systems, ion homeostasis, and second messenger-mediated signaling (Supplementary Figure S1). These results are consistent with known dysfunctions in CRC, suggesting that our results are reliable.

### WGCNA and identification of critical modules

**Figure 3:**
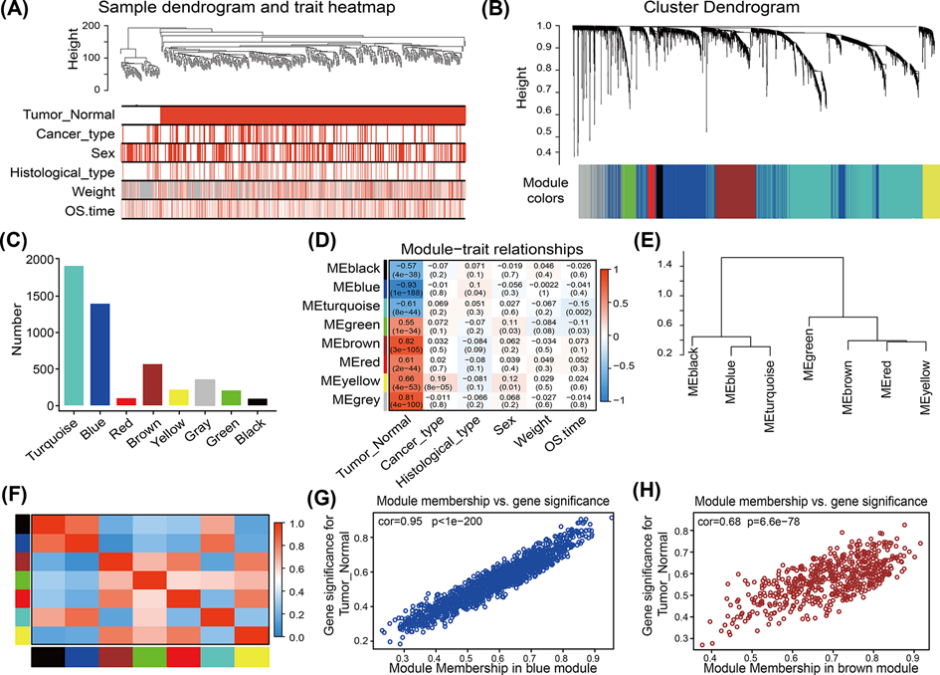
WGCNA of DEGs in CRC (**A**) Clustering dendrogram of the clinical traits and data from 420 CRC samples. Red color represents ‘tumor’ for the variable ‘Tumor_Normal’, ‘colon and rectum cancer’ for the variable ‘Cancer-type’, ‘female’ or ‘male’ for the variable ‘Sex’, and ‘adenocarcinoma’ or ‘mucinous carcinoma’ for the variable ‘Histological_type’. For the variables ‘Weight’ and ‘OS time’, red color is directly proportional to the value. (**B**) Dendrogram of 4832 DEGs depending on the dissimilarity measure 1-TOM (see ‘Materials and methods’ section). Each branch represents a gene, and each color represents a co-expression module. (**C**) Numbers of genes in the eight modules. (**D**) Heatmap of the correlation between module eigengenes (MEs) and clinical characteristics of CRC patients. Each cell contains the correlation coefficient and *P-*value. (**E,F**) Module eigengene dendrogram and heatmap of eigengene adjacency. (**G,H**) Scatter plots of GS score and MM (see ‘Materials and methods’ section) for genes in the (G) blue and (H) brown modules.

WGCNA was used to construct a network based on the expression matrix of 4832 DEGs and clinical data from 420 CRC samples. We performed cluster analysis to check the quality of the data from the 420 samples, all samples were in the clusters and within the cut-off threshold value (height < 200), therefore, no outliers were identified for removal (Figure 3A). Six clinical variables were applied in the WGCNA (Figure 3A): disease status (Tumor_Normal), cancer type, sex, histological subtype, body weight, and survival time (OS.time). The 420 samples fell into two clusters, Tumor and Normal.

To construct a scale-free network, we set the soft threshold power β to 7, the independence degree to 0.9, and the mean connectivity was close to 0 (Supplementary Figure S2A–D). DEGs with similar expression patterns clustered into the same modules, and modules showing a difference in cut height < 0.25 were merged. This procedure yielded eight co-expression modules: turquoise, blue, red, brown, yellow, gray, green, and black (Figure 3B,C, Supplementary Table S6). The gray module contained genes that could not be incorporated into any other module.

The eigengenes of the brown module strongly correlated positively with CRC (cor = 0.82, *P*=3 × 10^−105^), while the eigengenes of the blue module strongly highly correlated negatively with CRC (cor = −0.93, *P*=1× 10^−88^) (Figure 3D). These correlations were confirmed through analysis of hierarchical clustering, heatmaps, and adjacency relationships (Figure 3E,F). These results indicated that the brown module might contribute to tumorigenesis in CRC, while the blue module might protect against CRC. Therefore, the brown and blue modules were analyzed for hub genes.

### Identification of candidate hub genes from brown and blue modules

MM and GS scores strongly correlated positively with each other in the brown and blue modules (Figure 3G,H). The criteria for selecting hub genes relatively lower than the standard cut-off threshold (MM > 0.8). In the brown module, 151 genes were identified that satisfied the thresholds of ‘cor.gene ModuleMembership’ > 0.75 and ‘cor.geneTraitSignificance’ > 0.6. In the blue module, 150 genes were identified that satisfied the thresholds of ‘cor.geneModuleMembership’ > 0.75, and ‘cor.gene TraitSignificance’ > 0.7.

### Hub gene expression and correlation with survival

**Figure 4:**
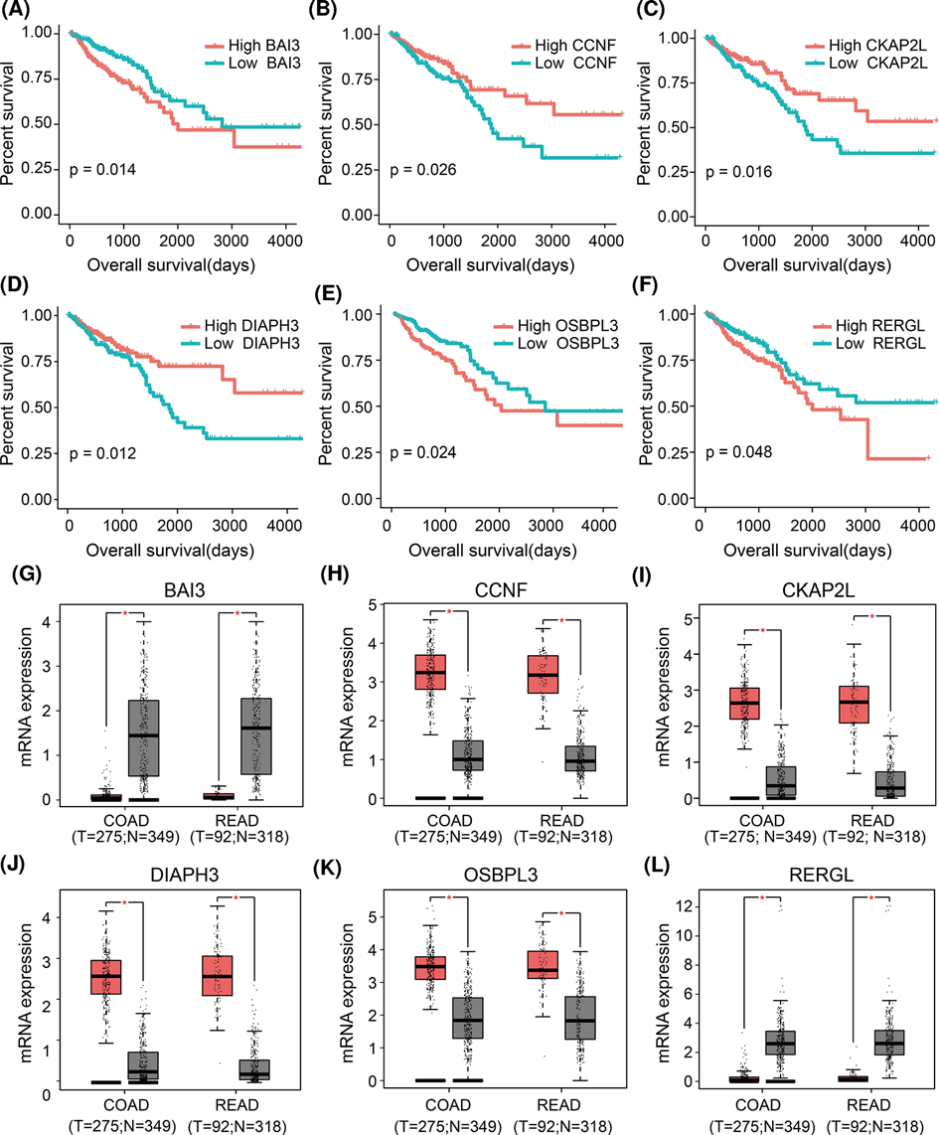
Survival analysis and validation of six hub genes using an independent dataset (**A–F**) Kaplan–Meier survival curves of CRC patients stratified by low or high expression of the six hub genes. (**G**–**L**) Differences in expression of the six hub genes between normal and tumor tissues in the GEPIA database. *P<0.01.

Based on expression data and clinical information for 360 CRC tumor samples in the TCGA, we examined potential associations between expression and patient survival for the 151 genes identified in the brown module and the 150 identified in the blue module. The brown module genes *CCNF, CKAP2L*, and *DIAPH3* were associated with prognosis, as were the blue module genes *BAI3, OSBPL3*, and *RERGL* (Figure 4A–F). Thus, we defined these genes as ‘final’ hub genes.

Using GEPIA website, we confirmed that the expression of all these hub genes were significantly different between normal and CRC tissues (Figure 4G–L). *BAI3* and *RERGL* were down-regulated in CRC, whereas the other hub genes were up-regulated. Similar results were obtained using data from the GEO database (Supplementary Figure S3A–F).

### Mutation landscape of hub genes

**Figure 5:**
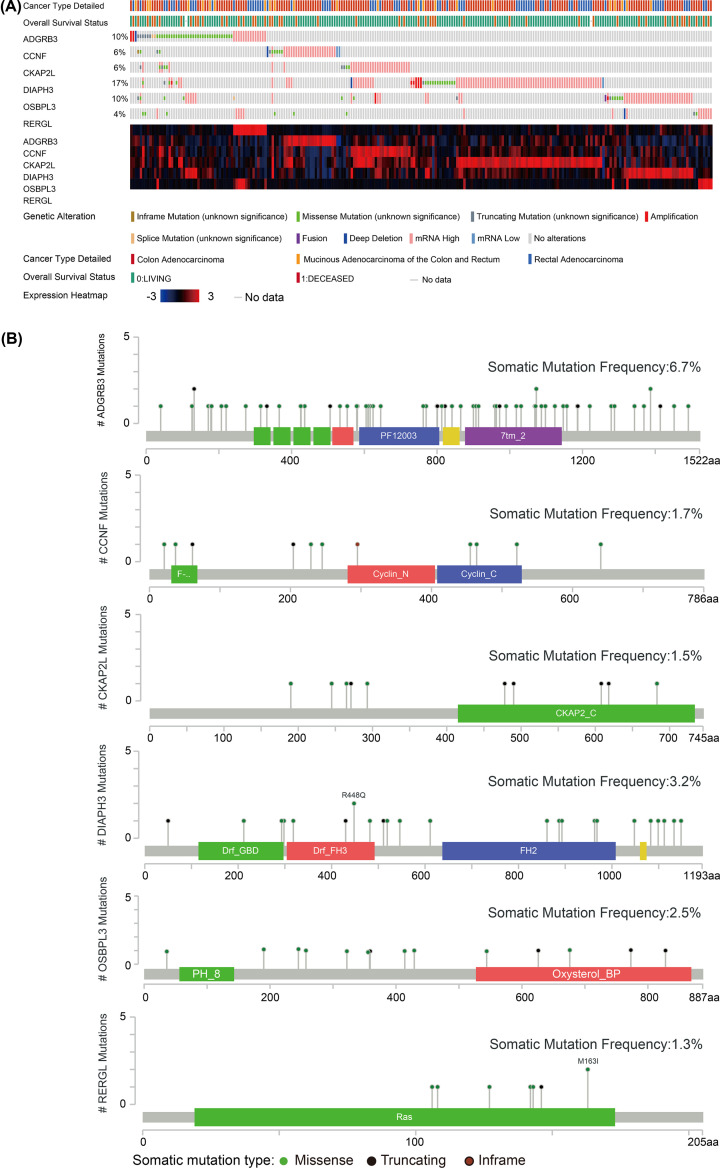
Mutations in the six hub genes, based on CRC data in TCGA (**A**) Bar plots and heatmaps showing mutations in the six hub genes. (**B**) Lollipop plots showing the distributions of mutations in different domains of the proteins encoded by the six hub genes.

The OncoPrint view of hub genes in the CBioPortal database was used to visualize mutations in the six hub genes based on data from 594 CRC patients in the TCGA. Nearly half of these patients (41%) had mutations in all six hub genes. The highest rate of mutations was observed for *DIAPH3* (17%), with missense mutations and mutations leading to higher mRNA expression being the most frequent (Figure 5A). *BAI3* showed the highest somatic mutation rate (6.7%), and the most frequent mutations were missense mutations and deletions (Figure 5B).

### Immunohistochemical validation of *OSBPL3* as a hub gene

**Figure 6:**
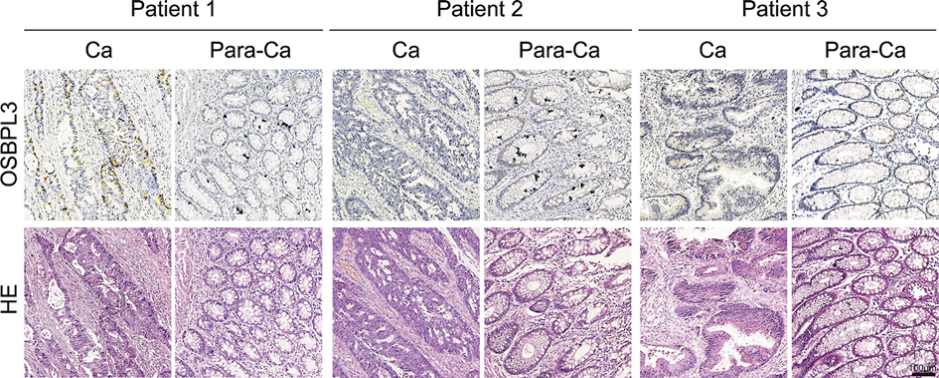
Different expression of *OSBPL3* in tumor tissues (Ca) and adjacent normal tissues (Para-Ca) Scale bar, 100 μm. Abbreviation: HE, Hematoxylin–Eosin.

We further validated the clinical significance of *OSBPL3* as a hub gene using immunohistochemistry (Figure 6). We detected that *OSBOL3* have heterogeneous expression in different types of tumor cells. *OSBPL3*, which localized mainly in the cytoplasm, was highly expressed in tumor cells and glandular epithelial cells, but less expressed in other cell types. Clearly indicate how its expression compared between tumor and normal samples overall.

## Discussion

CRC remains one of the world’s most malignant cancers. Although some studies have used WGCNA to explore molecular markers related to its pathogenesis, diagnosis and prognosis [34–36], the present work provided a more complete novel idea. We performed bioinformatics analyses across independent patient cohorts to identify biomarkers, one of which was experimentally validated. Our results suggest that poor prognosis in CRC is associated with overexpression of *CCNF, CKAP2L, DIAPH3* and *OSBPL3*, and with underexpression of *BAI3* and *RERGL*. Meanwhile, *BAI3* and *CKAP2L* may be novel prognostic markers for CRC.

Consistent with the predicted functional enrichment of our CRC DEGs genes, the cell cycle has been shown to be dysregulated in many types of cancer [37]. Many studies have shown that targeted regulation of cancer cell cycle is a potential treatment strategy [38]. Therefore, studying cell cycle pathway may advance the understanding of oncogenic mechanisms and the treatment options for CRC. Similarly, defects in DNA replication can lead to mutations, chromosomal poly- or aneuploidy, as well as gene copy number variations, all of which can lead to cancer [39]. DNA mismatch repair (MMR) deficiency is one of the most well-known forms of genetic instability in CRC [40].

Through WGCNA, we identified module and hub genes likely to be important in CRC. We determined two key modules, brown and blue, whose genes are strongly related to CRC (Tumor_Normal). Genes in the red, yellow, and turquoise modules from our analysis may also play roles in CRC. Therefore, our results indicate that complex gene networks regulate CRC occurrence and development. Six hub genes in the brown and blue modules strongly correlated with overall survival of CRC patients: *BAI3/ADGRB3, CCNF, CKAP2L, DIAPH3, OSBPL3*, and *RERGL*. Two of these, *BAI3* and *CKAP2L*, have not previously been linked to CRC, although expression of *BAI3*, a member of the BAI family [41], appears to be altered in malignant gliomas [42] and small cell lung cancer [43]. Similarly, expression of *CKAP2L*, a mitotic spindle protein, appears to be altered in lung adenocarcinoma [44], breast cancer [45], and non-small cell lung cancer [46].

The remaining four hub genes have previously been associated with CRC. *CCNF* is a founding member of the F-box family of proteins [47]. It can form the Skp1-Cul1-F-box protein ubiquitin ligase complex, which controls centrosome duplication and helps stabilize the genome [48]. Levels of *CCNF* can independently predict poor prognosis in patients with hepatocellular carcinoma [48]. Higher *CCNF* expression has been associated with longer survival in CRC [49]. *DIAPH3*, a formin ortholog [50], participates in actin remodeling and regulates cell movement and adhesion [51]. It can contribute to the development, invasion, and metastasis of lung adenocarcinoma, colorectal carcinoma [52–54]. *OSBPL3* participates in lipid metabolism, vesicle trafficking, and cell signaling [55,56], and it is up-regulated in malignancies such as Burkitt’s lymphoma and CRC [57]. *RERGL* is a tumor suppressor gene of the Ras superfamily, and its underexpression has been linked to overall survival in CRC [58,59].

The hub genes that we identified differ from those identified in previous studies of CRC-related genes. One study identified three novel hub genes that could be candidate genes for CRC molecular mechanism studies (*INHBA, CBX2*, and *BEST2*) [60], while another identified seven that may contribute to early onset of CRC (*SPARC, DCN, FBN1, WWTR1, TAGLN, DDX28*, and *CSDC2*) [61]. Other studies have focused on specific genes in CRC, such as *METTL3* [62] and *METTL14* [63]. Differences in the key genes detected across these various studies may reflect differences in the clinicopathological characteristics of patients and in the types of analyses performed. However, we used a combination of bioinformatics analysis, experimental verification and dataset cross-validation to study CRC-related DEGs, and obtained two novel hub genes (*BAI3* and *CKAP2L*) that may be associated with CRC’s prognosis. These results indicated that the present study provides new ideas for the study of molecular mechanisms of CRC.

Most previous WGCNA studies did not attempt to validate their genetic findings experimentally. Our six hub genes, which we validated in independent patient samples using bioinformatics and, in the case of *OSBPL3*, using immunohistochemistry, may help guide further studies to gain a comprehensive understanding of the network of genes involved in CRC. Such work may provide valuable clues for the treatment of CRC.

## Supplementary Material

Supplementary Figures S1-S3

Supplementary Tables S1-S6

## Data Availability

The data used in the present study was obtained via an online database. The GSE33113 dataset was collected from the GEO (https://www.ncbi.nlm.nih.gov/geo/) with additional datasets obtained from the The UCSC Xena website (https://xenabrowser.net/).

## Abbreviations

CRC: colorectal cancer
DEG: differentially expressed gene
GEO: Gene Expression Omnibus
GO: Gene Ontology
GS: gene significance
MM: module membership
PCC: Pearson correlation coefficient
TCGA: The Cancer Genome Atlas
TOM: topological overlap matrix
WGCNA: weighted gene co-expression network analysis
