# Supplementary figures and images for: Identification of hub genes in colorectal cancer based on weighted gene co-expression network analysis and clinical data from The Cancer Genome Atlas
Source: Biosci Rep. 2021 Jul 26;41(7):BSR20211280. doi: 10.1042/BSR20211280 (PMC8314434; doi:10.1042/BSR20211280)

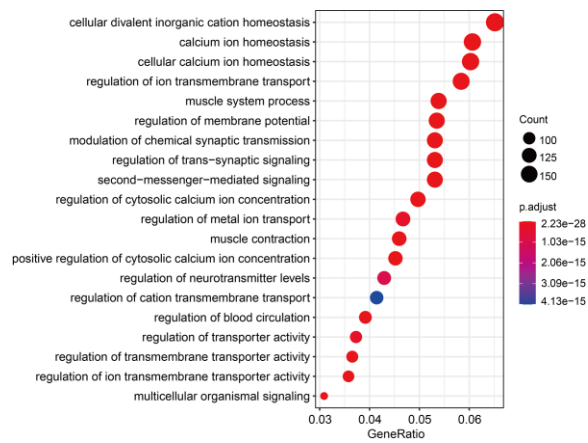

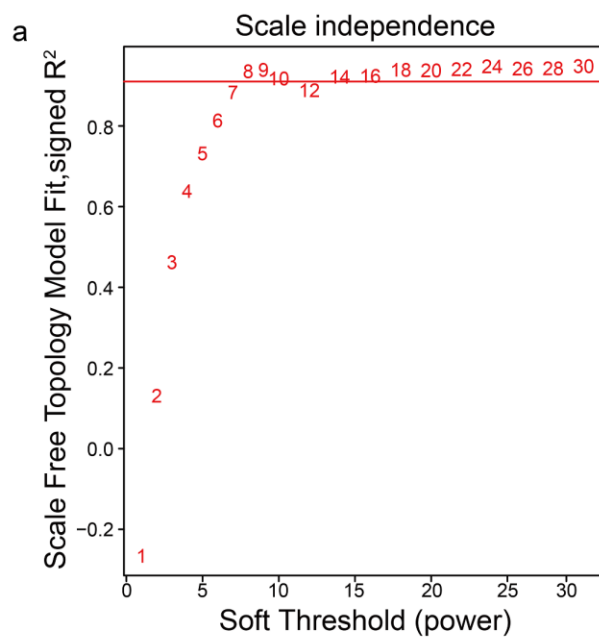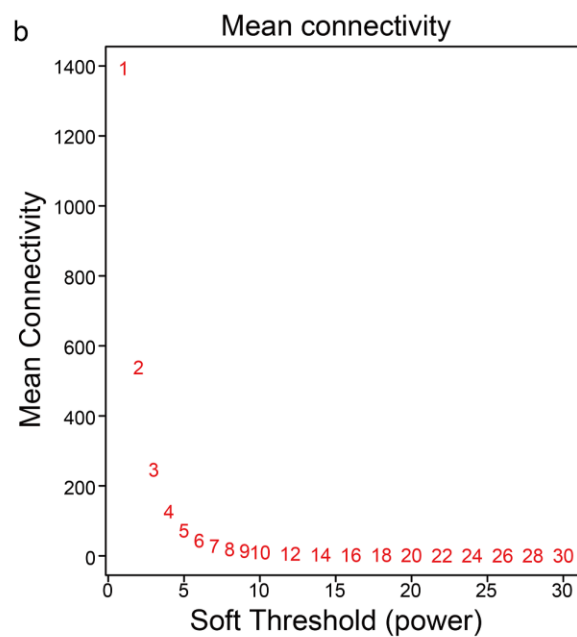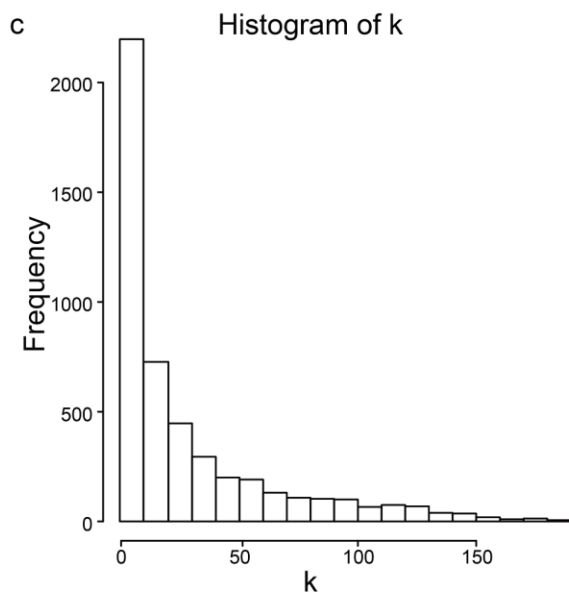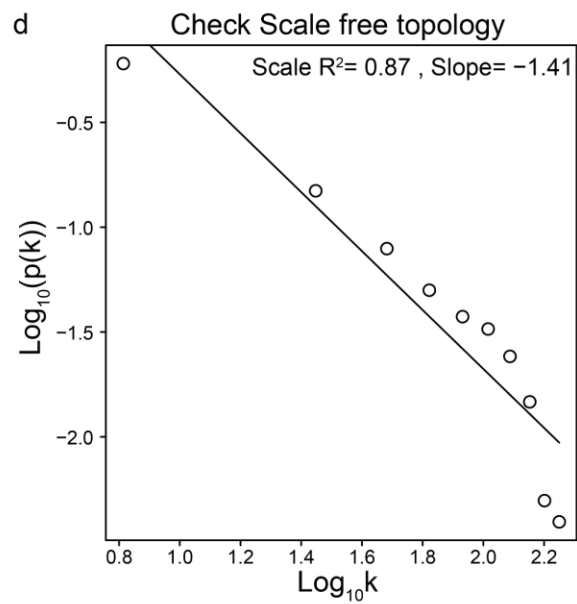

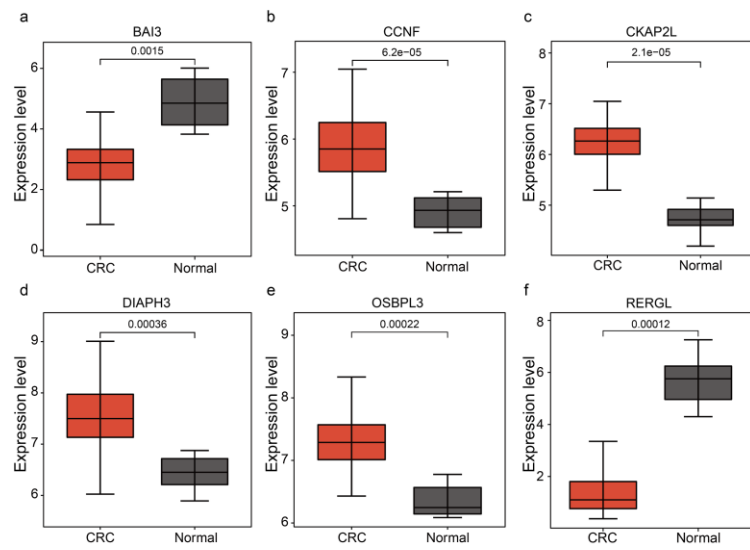

Supplement: Supplementary Figures S1-S3 [file BSR-2021-1280_supp.pdf]
